# Influence of the diameter of single-walled carbon nanotube bundles on the optoelectronic performance of dry-deposited thin films

**DOI:** 10.3762/bjnano.3.79

**Published:** 2012-10-17

**Authors:** Kimmo Mustonen, Toma Susi, Antti Kaskela, Patrik Laiho, Ying Tian, Albert G Nasibulin, Esko I Kauppinen

**Affiliations:** 1 NanoMaterials Group, Department of Applied Physics, Aalto University School of Science, P.O. Box 15100 (Puumiehenkuja 2), 00076 Aalto, Finland

**Keywords:** bundle diameter, sheet resistance, SWCNT, thin film, transmittance

## Abstract

The optoelectronic performance of thin films of single-walled carbon nanotubes (SWCNTs) was studied with respect to the properties of both individual nanotubes and their bundles. The SWCNTs were synthesized in a hot wire generator aerosol reactor, collected by gas filtration and dry-transferred onto various substrates. By thus completely avoiding liquid dispersion steps, we were able to avoid any artifacts from residual surfactants or sonication. We found that bundle lengths determined the thin-film performance, as would be expected for highly resistive bundle–bundle junctions. However, we found no evidence that contact resistances were affected by the bundle diameters, although they did play a secondary role by simply affecting the absorption. The individual SWCNT diameters and their graphitization level as gauged by the Raman D band intensity did not show any clear correlation with the overall performance.

## Introduction

Single-walled carbon nanotubes (SWCNT) offer great application potential in future electronics, such as micro-electromechanical devices [1], sensors [2–3], transparent electrodes [4–6], thin-film field-effect transistors [7–8] and capacitors [9]. However, most methods of fabricating devices rely on dispersion of the nanotubes in solutions. While the techniques are suitable for research, process-induced damage, such as tube cutting due to sonication [10–11], or residual surfactants severely limit device performance. Structural features such as SWCNT length, degree of bundling, and bundle length, diameter and orientation have received less attention, despite the fact that the electrical resistance of a SWCNT network is thought to be dominated by intertube and interbundle contact resistances [6,12]. Understanding the impact of these properties is crucial for gaining a fundamental understanding of the origins of optimal device performance and for the development of improved synthesis and deposition methods.

Synthesis and sample preparation techniques can have a strong impact on SWCNT network performance. Depending on the synthesis method, the nanotubes can have varying degrees of crystallinity, not to mention variation in the individual SWCNT and bundle length and diameter characteristics. Additional cleaning and purification steps can be detrimental for SWCNT network performance, especially in the case of sonication (cutting), acid cleaning (unintentional doping) or surfactant-based dispersions (residual surface contamination) [13–14]. Characteristic features have been explored previously by geometric scaling arguments and by comparing the performance of SWCNT films fabricated by different synthesis and sample-preparation routes. For example, Hecht et al. induced mechanical damage to liquid-suspended SWCNT bundles synthesized with the arc discharge and laser ablation methods, and were thus able to control the bundle lengths and diameters to some extent [14]. Geng et al. conducted a more thorough comparison between the performances of SWCNT networks from chemical vapor deposition (CVD), HiPCO, laser ablation, and arc discharge sources; although, again involving liquid suspensions [15]. While these initial studies have been steps in the right direction, the damage induced by sample preparation and the pervasive presence of surfactant material on the tubes limits the possibility to draw definite conclusions. More recently, Nirmalraj et al. (2009) worked with contact-mode atomic force microscopy (C-AFM) towards a direct measurement of the relation between bundle diameters and contact resistances [12]. However, due to their sample fabrication method it is likely that residual doping and surfactants were present in the samples, impacting on the results of their measurements.

By comparison, aerosol CVD synthesis offers a unique platform to study the impact of bundle characteristics on the performance of SWCNT networks, enabling direct deposition of highly pure and crystalline SWCNT bundles of varying length and diameter on a wide range of substrates. This renders the liquid dispersion unnecessary, enabling a clearer elucidation of the effects of bundle characteristics alone. The technique has yielded world-record-performance SWCNT films over a wide range of thicknesses from sub-monolayer networks for thin-film transistor channels [8], to high-performance, optically transparent electrodes [6,16]. Quite recently, both dry deposition of aerosol-synthesized tubes and SWCNTs from liquid dispersion have been shown to compete, and even exceed, the performance of indium tin oxide (ITO) coatings on plastic substrates in terms of optoelectronic performance [6,17]. Moreover, ITO has multiple additional drawbacks, including a high refractive index, spectrally nonuniform optical transmission, very limited flexibility, restricted chemical robustness, and most importantly, depleting raw material supply [18–19].

In this contribution, we focus on an investigation on the effects of the properties of individual SWCNTs and their bundles on the optoelectronic performance of SWCNT thin films, i.e., their network conductivity and absorption. We utilize a hot-wire-generator (HWG) [20] aerosol CVD reactor to fabricate films of SWCNTs with a wide range of bundle diameters and lengths using the dry deposition technique [6,16]. Also, a set of data from films previously fabricated in a similar manner by utilizing a ferrocene-based aerosol CVD reactor [21] is included for comparison. The bundle lengths are shown to dominate the optoelectronic performance, while bundle diameters play a secondary role by affecting the absorption. The diameters of the SWCNTs and their graphitization level do not seem to be important characteristics in our samples.

## Experimental

### Experimental setup

A hot wire generator (HWG) floating catalyst method was used to synthesize SWCNTs, which were subsequently utilized to fabricate SWCNT thin films. A complete description of the reactor design can be found elsewhere [22]. Briefly, iron particles were produced by vaporization from a resistively heated iron wire (current 2.7 A, diameter 250 μm, purity 99.95%, Goodfellow, USA) in a H_2_/Ar (7/93 mol ratio) flow of 480 cm^3^·min^−1^ inside an aluminium oxide (Al_2_O_3_) tube (inner diameter 16 mm). Particles formed and grew through vapor nucleation–condensation and coagulation processes inside an Al_2_O_3_ tube reactor (inner diameter 22 mm) and were mixed with 500 cm^3^·min^−1^ CO, together with 1300 ppm of CO_2_, in the heated section of the furnace. The mixing zone resided 29 cm from the bottom inlet of the reactor, corresponding to wall temperatures of 460–700 °C. The aerosol concentration was monitored at the reactor outlet with a GRIMM Vienna Type differential mobility analyzer (DMA) and a Faraday cup electrometer (SMPS+E). The catalyst source was fixed in a temperature zone in which carbon nanotube growth is known to be possible [23], corresponding to a wall temperature of 700 °C (at a depth of 29 cm). The position of the HWG was kept constant, while the furnace temperature (*T*_set_) was varied.

#### Sample preparation

Samples of SWCNTs with different characteristics were synthesized by systematically varying the furnace set temperature, *T*_set_. The true maximum furnace temperature was 10 to 15 °C higher than *T*_set_, which ranged from 550 to 800 °C at 50 °C intervals. The CO_2_ concentration was kept constant at 1300 ppm. The wire current of the HWG, affecting the catalyst number concentration through the evaporation rate, was kept constant at 2.7 A. In addition, a benchmark sample was synthesized under previously optimized conditions at 880 °C with the introduction of 1500 ppm CO_2_. The SWCNTs were collected from the gas phase by filtering the flow at the reactor outlet through 10 mm diameter nitrocellulose disk filters (Millipore, HAWP, 0.45 µm pore diameter). In addition to the HWG samples produced in this contribution, a set of data from similarly fabricated films reported earlier was included in comparisons of the film properties [6,24]. The synthesis parameters along with the SWCNT and bundle characteristics are listed for all of the samples in Table 1.

**Table 1:** Properties of SWCNT films fabricated in this study, along with four datasets from the literature.

Method	*T*_set_ (°C)	*I*_G_/*I*_D_	*d*_bundle_ (nm)	*L*_bundle_ (µm)	*d*_tube_ (nm)	*K* (kΩ^−1^)	*K*_NORM_ (µm·kΩ)^−1^

HWG	550	5.2	—	—	≈1.0	—	—
HWG	600	5.2	—	—	≈1.0	—	—
HWG	650	7.6	3.1 ± 1.0	0.17 ± 0.01	0.95 ± 0.10	2.4 × 10^−4^	2.7 × 10^−3^
HWG	700	22.4	3.4 ± 1.0	0.45 ± 0.05	1.07 ± 0.24	0.3	1.43
HWG	750	22.0	3.2 ± 1.5	1.13 ± 0.50	1.13 ± 0.26	2.2	3.24
HWG	800	33.5	5.2 ± 3.7	4.56 ± 0.80	1.60 ± 0.73	6.8	3.11
HWG	880	48.5	5.3 ± 2.5	9.80 ± 3.40	1.39 ± 0.40	14.0	3.09
HWG^a^	890	—	6.0 ± 3.0	3.00 ± 1.10	1.40 ± 0.30	5.2	4.06
FC^b^	880	—	8.3 ± 3.5	1.30 ± 0.80	1.95 ± 0.25	0.9	1.67
FC^b^	880	—	7.8 ± 2.7	3.30 ± 1.40	1.95 ± 0.25	2.8	1.98
FC^b^	1050	—	12.8 ± 4.1	9.40 ± 1.40	2.18 ± 0.35	9.8	3.08

^a^Previously published data for HWG aerosol CVD [6], ^b^Previously published data for ferrocene aerosol CVD [24].

#### Characterization

The as-deposited SWCNT networks were press transferred [6,16] from the low adhesion filters onto silicon or aluminium substrates for scanning electron microscope observation (SEM, JEOL JSM-7500FA, Japan) used to measure the SWCNT bundle lengths (*L*_bundle_). Similarly, SWCNT networks were transferred onto optically transparent 1 mm thick quartz substrates (HQS300, Heraeus) for Raman spectroscopy (LabRAM, HORIBA JobinYvon, France) utilizing a HeNe laser source of 632.82 nm (1.96 eV), and for UV–vis–NIR absorption spectroscopy (Perkin-Elmer Lambda 950) used to determine the SWCNT diameters (*d*_tube_). For high-resolution transmission electron microscope observation (HRTEM, double aberration-corrected JEOL JEM-2200FS) of the SWCNT bundle diameters (*d*_bundle_), a similar dry-transfer approach was implemented. Copper grids with holey-carbon coating were manually pressed against quartz substrates with the SWCNT networks, transferring a near-monolayer of SWCNTs onto the grids. The sheet resistances (*R*_s_) were recorded with a four-point probe and a RM3-AR Test Meter (60 ± 5 g loading, Jandel Engineering, UK) from the SWCNT networks transferred onto quartz substrates.

## Results

**Figure 1:**
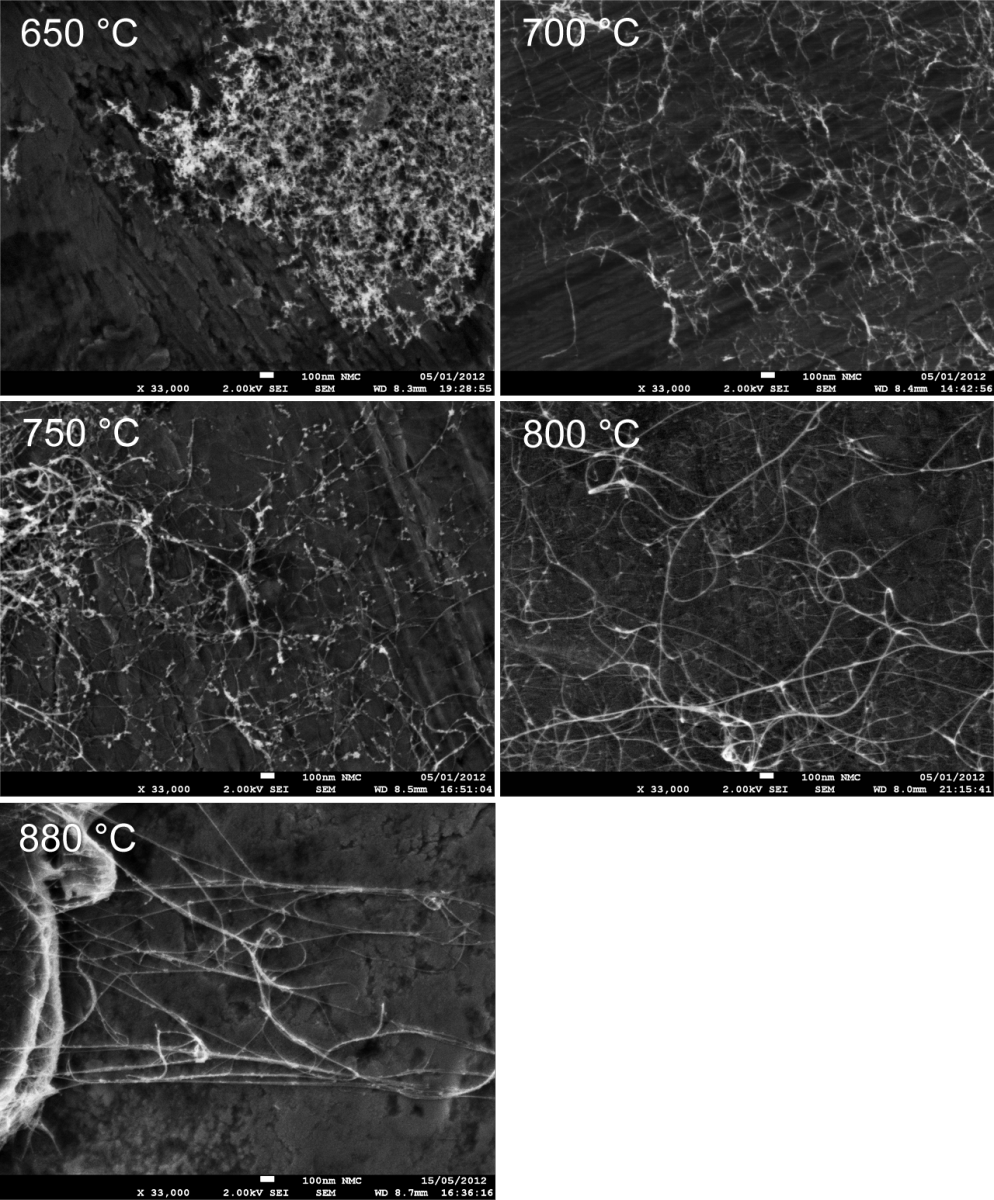
SEM images of as-prepared SWCNT networks dry-transferred onto the aluminium substrate. From top left, synthesis temperature increases from 650 to 880 °C and the average bundle length from 0.17 ± 0.01 to 9.80 ± 3.40 µm.

**Figure 2:**
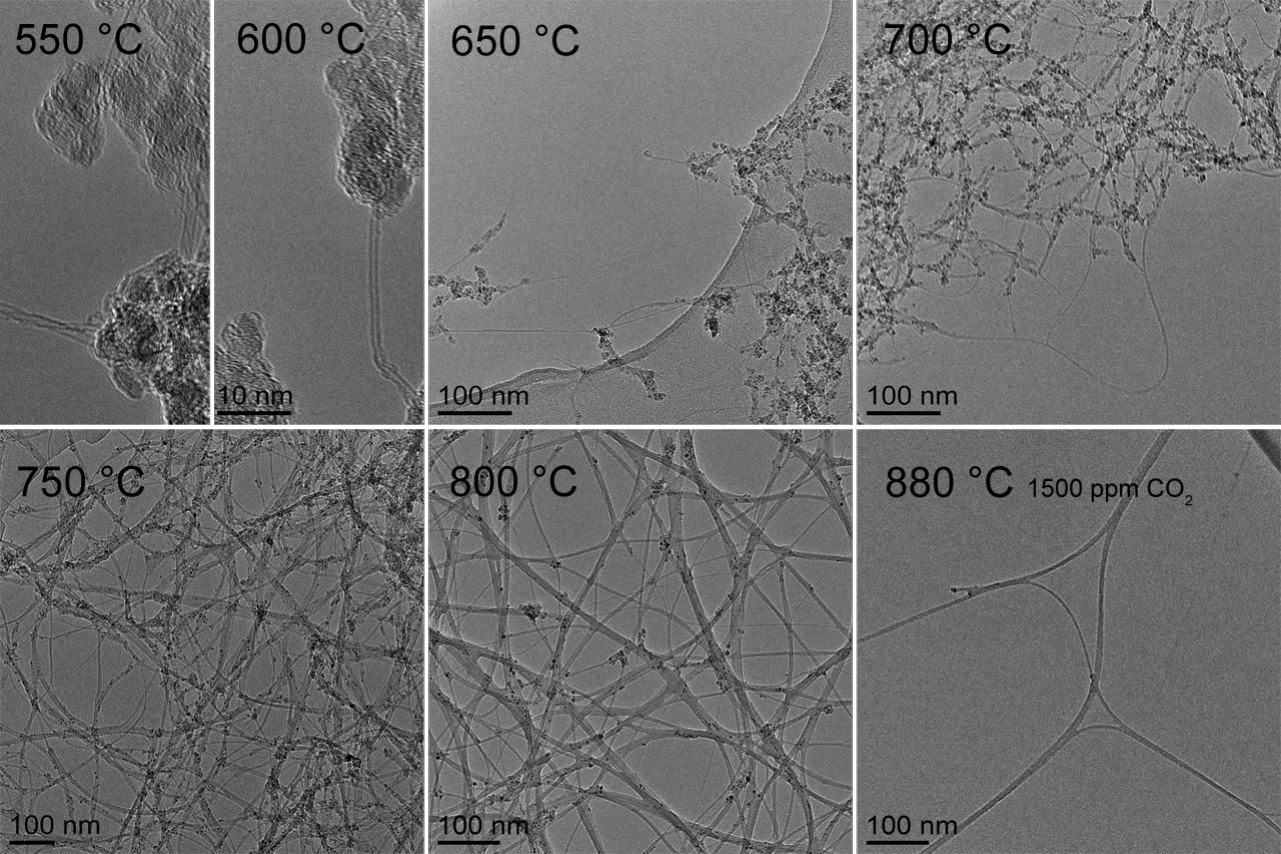
TEM micrographs of the as-produced SWCNTs. The synthesis conditions and corresponding bundle diameters are 550 °C, 1300 ppm, N/A; 600 °C, 1300 ppm, N/A; 650 °C, 1300 ppm, 3.1 ± 1.0 nm; 700 °C, 1300 ppm, 3.4 ± 1.0 nm; 750 °C, 1300 ppm, 3.2 ± 1.5 nm; 800 °C, 1300 ppm, 5.2 ± 3.7 nm; and 880 °C, 1500 ppm, 5.3 ± 2.5 nm. Note that the 880 °C sample was collected electrostatically, resulting in a lower SWCNT density on the TEM grid.

**Figure 3:**
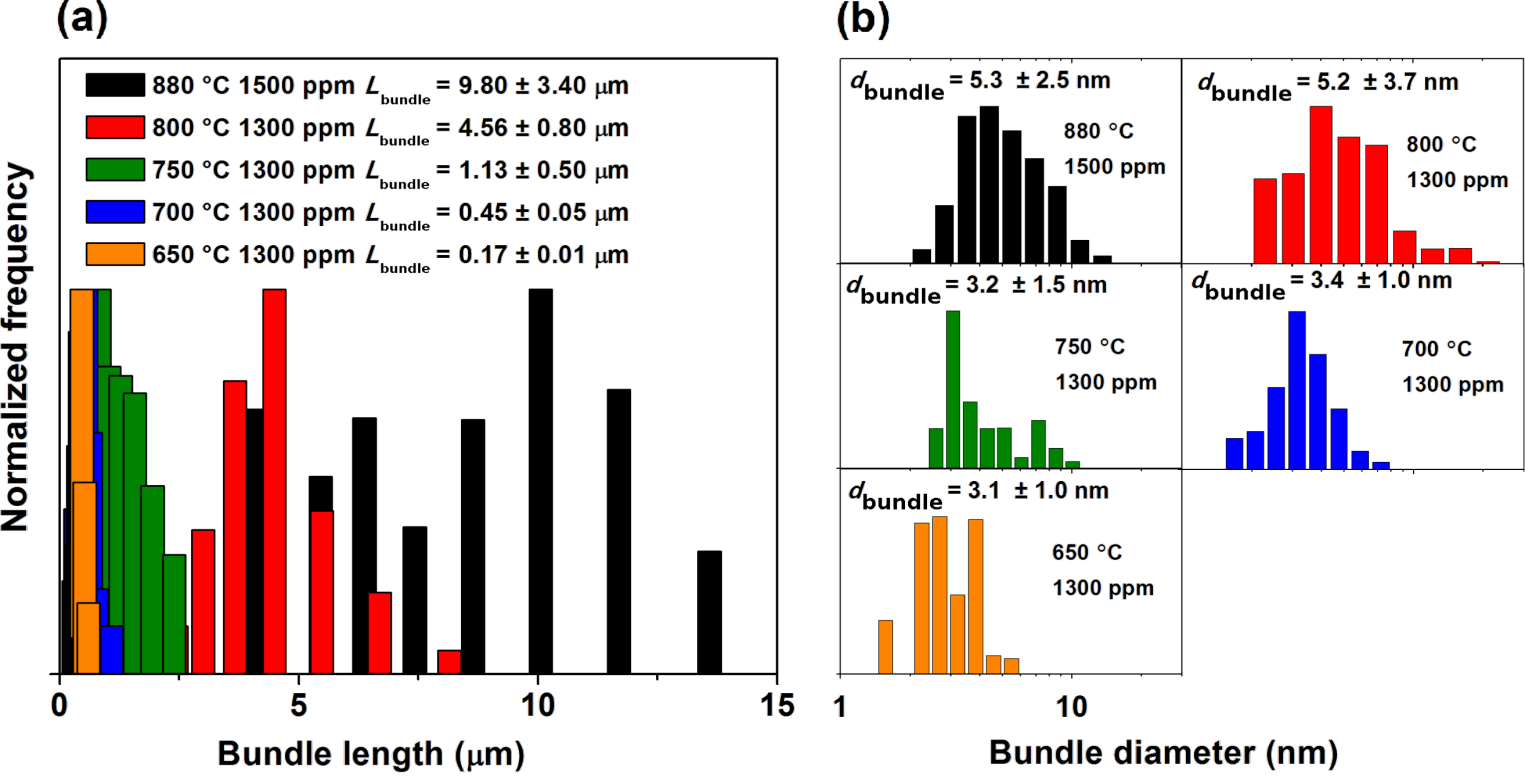
(a) Bundle length statistics measured from SEM images, and (b) bundle diameter statistics from TEM micrographs.

Varying the synthesis temperature (*T*_set_) resulted in major changes in the overall network and bundle characteristics of the as-prepared SWCNTs, as was observed by SEM and TEM. The overall amount of amorphous impurities in the SWCNT networks reduced dramatically with increasing *T*_set_, as can be clearly observed in Figure 1. At *T*_set_ = 650 °C, the SWCNT networks were covered under a nearly continuous layer of amorphous carbonaceous material. At lower *T*_set_’s of 550 and 600 °C, the SWCNT networks were too sparse to form continuous areas, or to even be clearly visible in SEM. Even so, TEM observations revealed the existence of short (<100 nm) SWCNTs embedded in an apparently amorphous carbon sheet, as shown in Figure 2. The relative amount of this amorphous material visibly reduced as *T*_set_ was increased to 700 °C, and the bundles were also much longer (*L*_bundle 700 °C_ = 0.45 versus *L*_bundle 650 °C_ = 0.17 µm). The same trend was found to hold at higher *T*_set_ as well; *L*_bundle 750 °C_ = 1.13 µm, *L*_bundle 800 °C_ = 4.56 µm, and *L*_bundle 880 °C_ = 9.80 µm. The corresponding *L*_bundle_ distributions are presented in Figure 3a. Both the decrease in the relative amount of amorphous material and the increase in *L*_bundle_ were expected, since the catalytic activity of iron nanoparticles and diffusion rate of carbon are both more suitable for SWCNT production at higher temperatures [25–26]. This was also clearly evidenced by an increase in the reactor output concentration as confirmed with the DMA measurements: the number concentration (*NC*) increased steadily from *NC*_650 °C_ = 6 × 10^5^ cm^−3^ at *T*_set_ = 650 °C, with the corresponding geometric mean diameter *GMD*_650 °C_ = 45 nm, reaching *NC*_800 °C_ = 2 × 10^7^ cm^−3^ and *GMD*_800 °C_ = 55 nm at *T*_set_ = 800 °C. TEM observations also revealed an increase in the bundle diameters *d*_bundle_ with increasing *T*_set_, as depicted by the *d*_bundle_ distributions shown in Figure 3b.

Furthermore, SWCNTs synthesized at *T*_set_ < 650 °C were less able to withstand the electron irradiation dose in the TEM, disintegrating at magnifications higher than 600k. Their graphitization level, e.g., the crystallinity of the hexagonal carbon lattice, was thus likely poor, with the tubes containing a high concentration of defects. Therefore, in order to judge the relative SWCNT quality, we utilized resonant Raman spectroscopy. For us the most interesting features in the Raman spectra were the intensities of the G and D bands, along with the radial breathing modes (RBM). In graphitic carbon, the G band (~1580 cm^−1^) corresponds to planar vibrations of carbon atoms, while the D band (~1350 cm^−1^) is sensitive to structural defects and impurities such as amorphous carbon and vacancies in the sp^2^-hybridized carbon lattice [27]. Therefore, the ratio of the intensities of the G and D bands (*I*_G_/*I*_D_) in the Raman spectra was used as a measure of the material graphitization level as a whole. RBMs (about 100–350 cm^−1^), on the other hand, correspond to the radial expansion–contraction of the SWCNTs, and their frequencies ω_RBM_ are correlated with SWCNT diameters *d*_tube_ by

**Figure 4:**
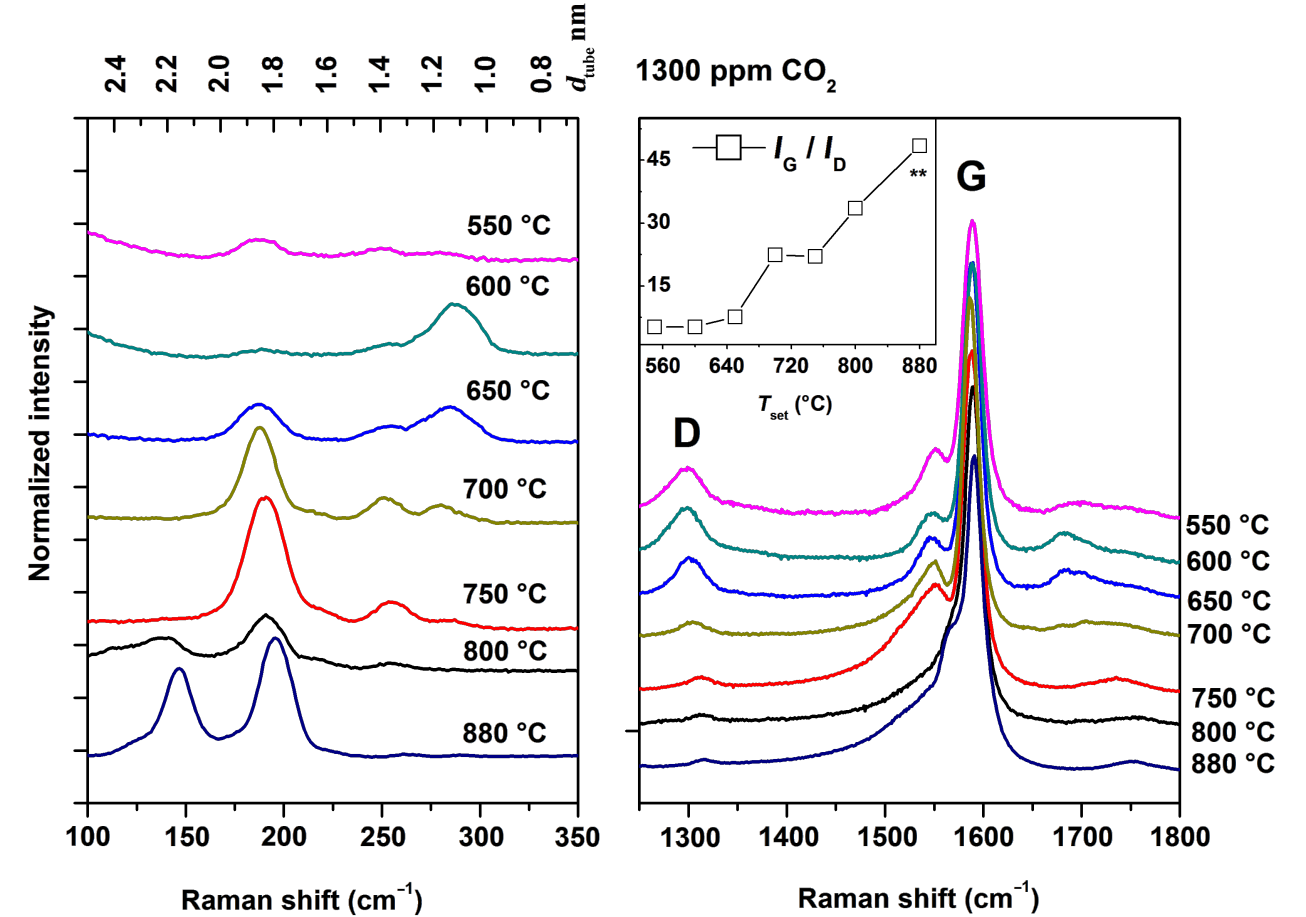
RBM modes (left) and D and G bands (right) of resonant Raman spectra recorded from SWCNTs synthesized under different temperature conditions (550–880 °C). The inset plots the ratio of G and D band intensities (*I*_G_/*I*_D_) with increasing *T*_set_. Laser wavelength of the Raman system was 632.82 nm (1.96 eV).

[1]
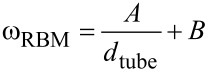


where *A* and *B* are determined experimentally [28]. Here, parameter values *A* = 248 cm^−1^ nm and *B* = 0 cm^−1^ were used to infer the diameters of SWCNTs in resonance with the laser excitation. These Raman features are shown in Figure 4.

The *I*_G_/*I*_D_ ratio for SWCNTs synthesized at *T*_set_ = 550 and 600 °C was roughly 5, which indicates either a very high defect density or a high impurity level. Certainly, the D band originates partly from the amorphous impurities on the samples, which were clearly seen by SEM and TEM. Regardless of the exact origin of the D band in our samples, a comparison of the *I*_G_/*I*_D_ ratio of samples from *T*_set_ = 650–880 °C revealed an increase of *I*_G_/*I*_D_ from 5.2 (550 °C) to 48.4 (880 °C). This indicates a substantial enhancement of sample quality with increasing *T*_set_ (cf. Figure 2 and Figure 4 inset).

**Figure 5:**
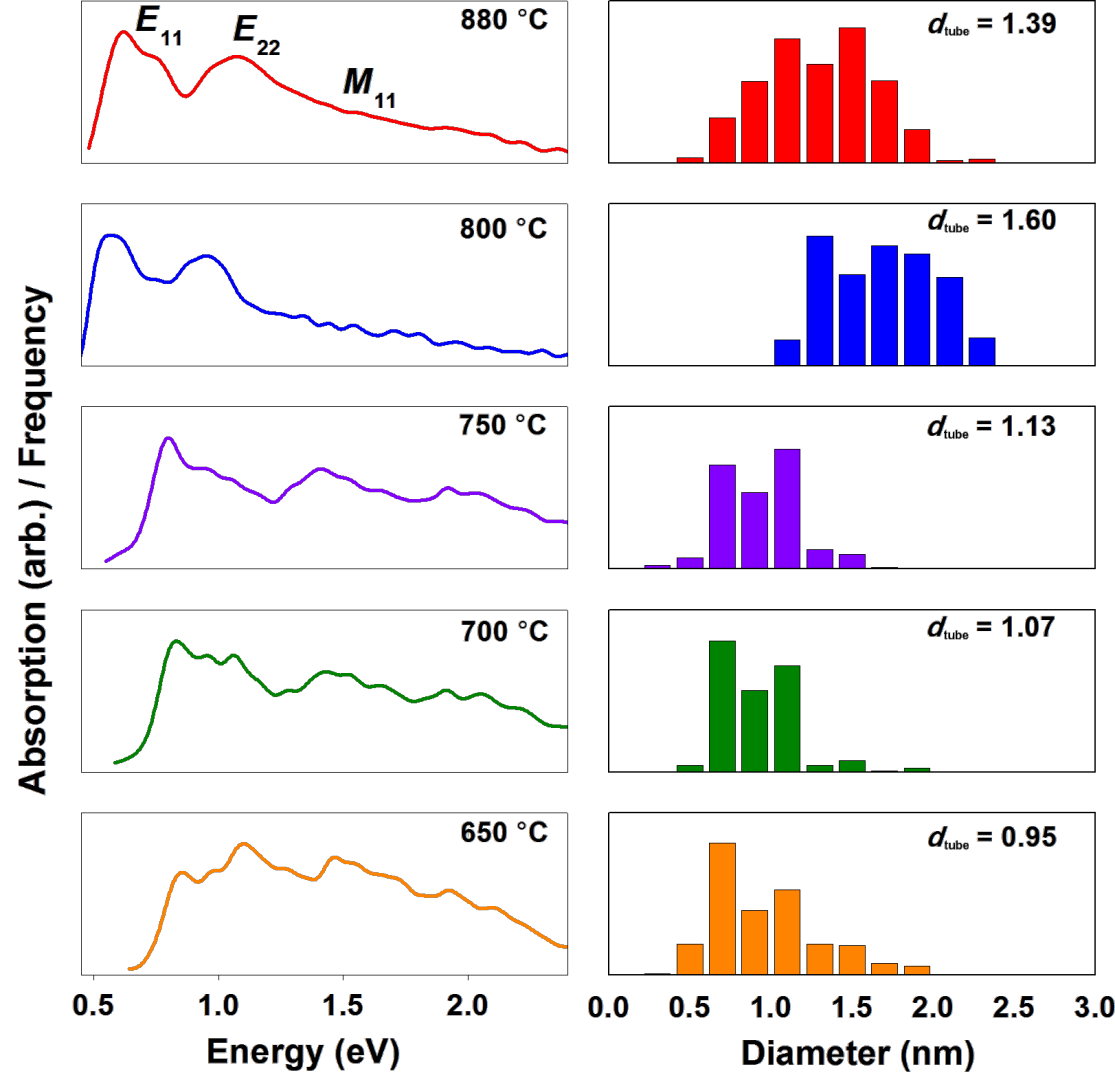
UV–vis–NIR absorption spectra (left) from the SWCNTs synthesized under different temperature conditions (650–880 °C) and the SWCNT diameter distributions (right) fitted to each spectrum.

In addition to SWCNT bundle characteristics and defect density, the SWCNT chirality distribution and diameters (*d*_tube_) can contribute both to conductivity and light-absorption properties [27,29]. UV–vis–NIR absorption spectroscopy is a versatile tool that can be used to define both *d*_tube_ and, to a limited extent, metallicity [30]. The fitting of semiconducting (*E*_11_ and *E*_22_) and metallic (*M*_11_) optical-transition peaks to the absorption spectra shown in Figure 5 resulted in the corresponding *d*_tube_ distributions plotted below the spectra. The mean *d*_tube_ increased with *T*_set_ from 0.95 nm (650 °C) to 1.60 nm (800 °C). However, the benchmark sample with *T*_set_ = 880 °C had a slightly smaller *d*_tube_ at 1.39 nm.

Finally, we consider the optoelectronic properties of the films. For networks that share the same total concentration of carbon (and thus absorbance) and general morphology, it was previously shown that the number of bundle–bundle contacts scales inversely with the average bundle length [24]. Since the network resistance scales linearly with the number of contacts, conductance thus scales linearly with the average bundle length. The absorbance (*A*) and conductance (σ_DC_) can be linked by the so-called figure of merit *K* [6]

**Figure 6:**
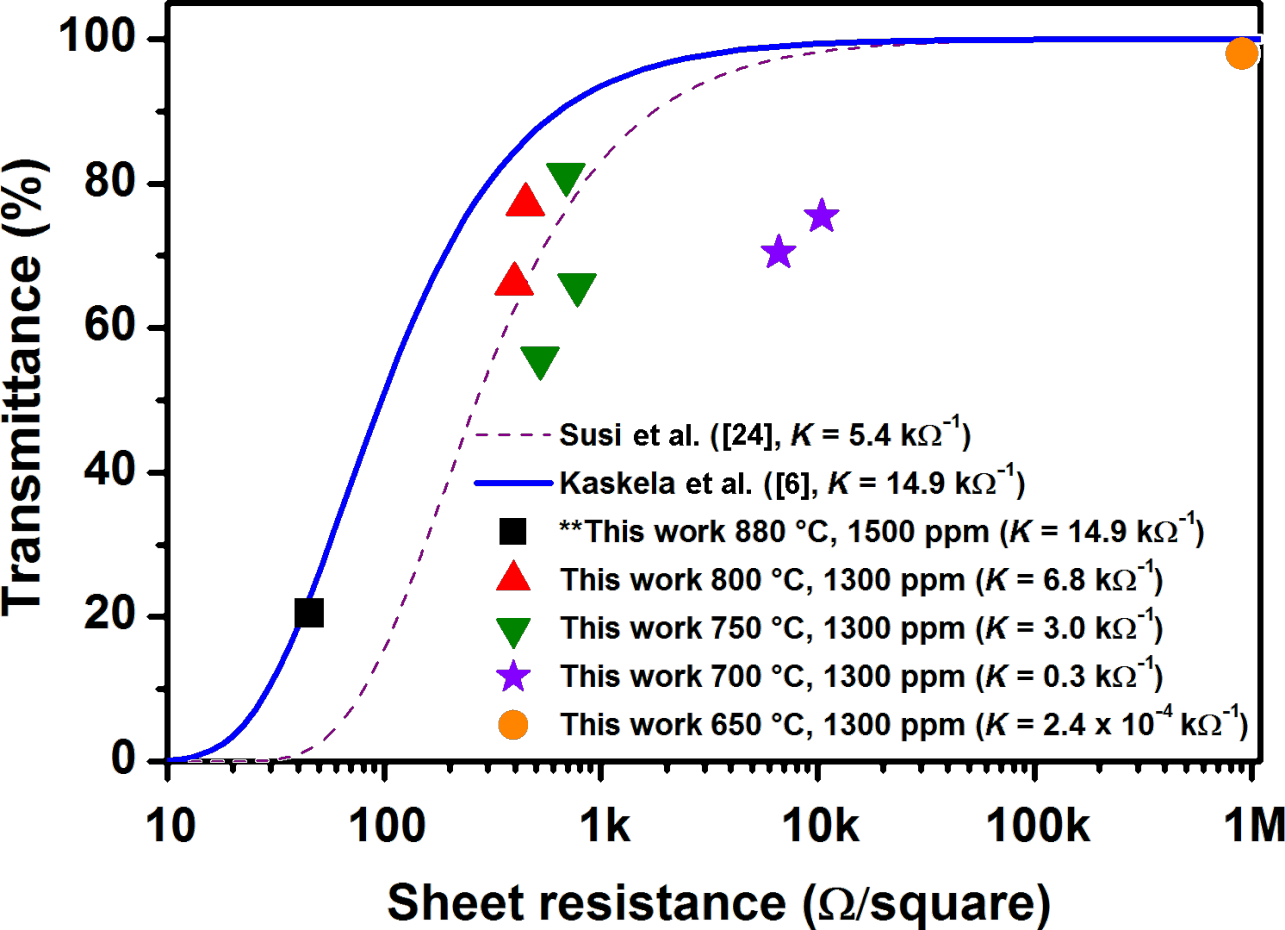
Comparison of sheet resistance versus optical transparency (at 550 nm) of SWCNTs synthesized at different temperatures (650–880 °C). The black square represents the benchmark sample (880 °C), red upright triangles SWCNTs from 800 °C, green downright triangles SWCNTs from 750 °C, purple stars SWCNTs from 700 °C and the orange circle SWCNTs from 650 °C. The blue solid line and the purple dashed line represent the literature data used for comparisons.

**Figure 7:**
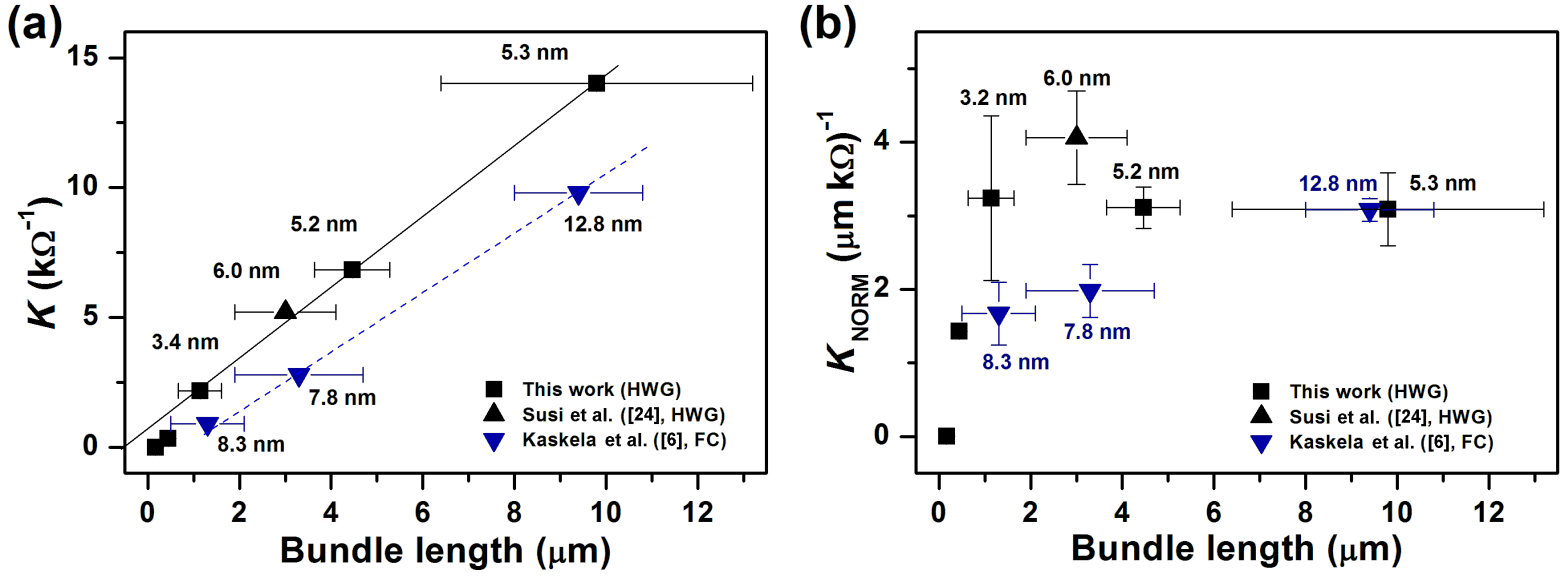
(a) The figure of merit *K* versus bundle length. A linear dependence of *K* on *L*_bundle_ is observed, as expected from geometric scaling arguments combined with the Beer–Lambert law. The higher the *K*, the better the optoelectronic performance. (b) Normalized figure of merit *K*_NORM_ versus *L*_bundle_. The apparent difference between the two datasets (black and blue) is greatly reduced.

[2]
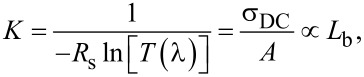


where *R*_s_ is the sheet resistance and *T*(λ) (later simply *T*) the transmittance measured at the middle of the visible spectrum (λ = 550 nm, or 2.3 eV). The UV–vis–NIR absorption spectra and four-point sheet resistance values shown in Table 1 were used to calculate the corresponding *K*’s shown in Figure 6. The solid curves represent previously published data for SWCNTs synthesized both by using a ferrocene [6] and a HWG aerosol CVD [24], while the scattered data represent the current study. The higher the figure of merit *K* is, the further left are the data situated in the plot. Referring to Equation 2, we may distinguish two distinct populations of SWCNTs when *K* is plotted as a function of *L*_bundle_, both following linear regression but with diverging slopes (Figure 7a). The continuous black line corresponds to SWCNTs from HWG aerosol CVD, while the blue dashed line corresponds to ferrocene aerosol CVD SWCNTs.

To elucidate contributions to film performance caused by nongeometric factors (i.e., junction resistances or contact barriers), the data can be normalized by eliminating the contributions of variations in bundle diameter and length.

According to the Beer–Lambert law, for films of a given thickness (*d*), absorbance (*A*) depends linearly on the concentration (*C*) of absorbers in the film (in our case carbon, *C*_carbon_). In carbon nanotube thin films, the carbon is distributed in the form of carbon nanotubes with an average tube length and diameter (*d*_tube_). Due to van der Waals interactions, the carbon nanotubes form regular bundles with an average length (*L*_bundle_) and average diameter (*d*_bundle_). We assume that the shape of a bundle is approximately independent of its constituent nanotubes, meaning that the length of a bundle is determined by the length of its nanotubes, and its diameter by the diameter of its nanotubes (*d*_tube_) and the degree of bundling caused by the synthesis process.

The total concentration of carbon on a given area (or volume) may then be expressed with the average areal (or volumetric) density (ρ_bundle_), the average length (*L*_bundle_) and the average diameter of bundles (*d*_bundle_). Typically the junction resistance, *R*_J_, is thought to be much higher than the intratube (or intrabundle) resistance, *R*_I_, that is*, R*_J_* >> R*_I_. Thus, the conductance of a film (σ_DC_) is expected to depend on the geometric parameters, both because shorter bundles will result in more high-resistance junctions per unit length, and possibly because the bundle geometry alters the resistive properties of the junctions.

**Figure 8:**
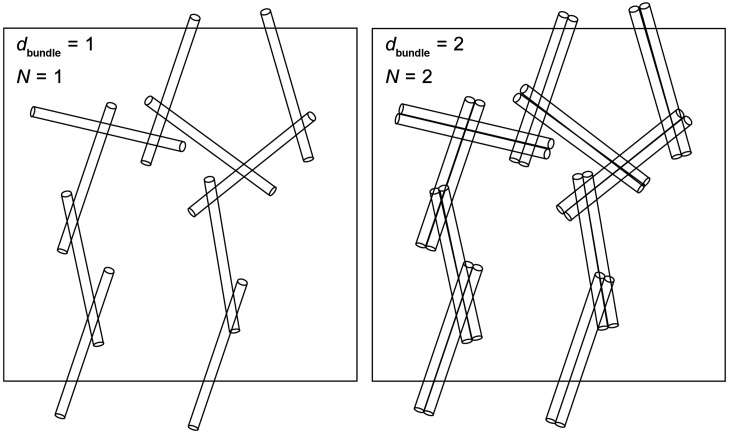
An illustration to clarify how the total concentration of carbon depends on bundle diameter for a given network morphology. Note that the number of bundle–bundle junctions is equal in both cases.

Given that the average bundle length, *L*_bundle_, and the average number density of bundles in the network, ρ_bundle_, remain constant, the average bundle diameter, *d*_bundle_, dictates the total concentration of carbon. This is best depicted by the illustration in Figure 8, graphically relating *C*_carbon_ and *d*_bundle_.

Obviously, *C*_carbon_ increases linearly with the average number of individual SWCNTs in bundles (*N*), which in the two-dimensional illustration depends linearly on *d*_bundle_. In reality, of course, the bundles are not flat but three-dimensional, and on average, circular in cross section. In three dimensions, we can evaluate *N*, and thus the total concentration of carbon, as

[3]
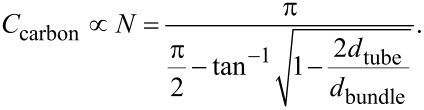


Equation 3 gives the average number of tubes per bundle in the case of circular bundles, containing more than three individual tubes. In our case, the average bundle diameters are systematically more than 3 nm (and *N* > 5), and thus, Equation 3 gives a good approximation for *N*. As was discussed earlier, absorbance is linked to the total number of individual absorbers and to *N* as well. Therefore it is justified to interpret *N* as a measure of absorbance induced purely by geometric effects, i.e., changes in bundle diameters.

Hence, for a set number of bundle–bundle contacts, the concentration of absorbers (*C*_carbon_), and thus film absorbance, scales linearly with the average number of individual SWCNTs in bundles (*N*). This can in turn be estimated from the average bundle diameter *d*_bundle_ using simple arguments introduced above. Therefore, we can normalize *K* taking into account both of these purely geometric effects, resulting in

[4]
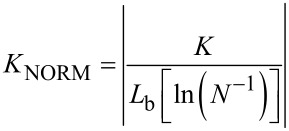


Figure 7b plots *K*_NORM_ as a function of *L*_bundle_, chosen as the *x*-axis since it is the most prominent factor defining the value of *K*. An immediate observation is that both *K* and *K*_NORM_ fall to zero slightly before *L*_bundle_ approaches zero. The physical interpretation of such behavior is the existence of a percolation threshold (e.g., the threshold where the long range connectivity of remote parts in a random network is lost), which becomes evident when *L*_bundle_ is very small. At the edge of the threshold, the conductivity of the network rapidly collapses.

Provided *L*_bundle_ strictly dictates the geometric scaling of conductance and *d*_bundle_ the geometric scaling of absorbance, the normalized values should lie on a vertical line, *K*_NORM_ = constant. Indeed, besides the points close to the percolation threshold (*L*_bundle_ < 1 µm), the normalized data are around *K*_NORM_ = 3 ± 1 µm^−1^·kΩ^−1^. Some small variation does remain: ferrocene aerosol CVD data (blue downward triangles) generally fall slightly below the average, while HWG aerosol CVD data (black squares and an upward triangle) appear slightly above the average, although in a much less pronounced manner than in the non-normalized data in Figure 7a. This suggests that the difference between the two datasets in Figure 7a is mostly due to the enhanced absorbance of the thicker ferrocene reactor bundles.

## Discussion

So far only the effect of the average bundle diameter on the absorbance has been considered. As was discussed, this entirely geometric effect can be taken into account by the normalization steps. Thus, any remaining effects are likely electronic in nature, possibly caused by modifications of the resistive junctions. The bundle diameters were effectively tuned by varying the synthesis temperature, which resulted in different aerosol number concentrations in the reactor. Larger diameter bundles are expected to form at higher number concentrations, which was indeed observed by comparing the reactor outlet aerosol concentrations with the average bundle diameters: higher concentrations resulted in larger bundles. Obviously, no significant correlation between the average bundle diameter and *K*_NORM_ can be observed in Figure 7b. Thus, it seems that *d*_bundle_ is responsible for changes in the optoelectronic performance of the films only as far as absorption is concerned.

This contradicts earlier work by Nirmalraj et al. [12], who used contact mode atomic force microscopy to determine the junction resistances of individual SWCNTs and bundles. Their data showed an approximately 10-fold increase in the junction resistances when the average bundle diameter increased from 3.5 ± 1.5 to 10.5 ± 4.9 nm. For such a large increase in junction resistances, the corresponding change in *K* and *K*_NORM_ would be approximately 10- and 20-fold, respectively. Our data shows practically no effect for a similar change of *d*_bundle_, as is evident in Figure 7a and Figure 7b. More work on direct measurement of the bundle–bundle contact resistances is clearly needed to resolve these discrepancies.

The SWCNT diameter *d*_tube_ modifies the electronic transitions of SWCNTs, and thus impacts not only the contact and Schottky barriers [31], but also the light-absorption properties [27,29]. SWCNTs of small *d*_tube_ have their first-order metallic-transition peaks (*M*_11_) located around 550 nm (2.3 eV), whereas for larger *d*_tube_ SWCNTs the peaks are shifted to lower energies (higher wavelengths). This slightly lowers the absorbance at the reference wavelength. Furthermore, contact and Schottky barriers are larger for SWCNTs of smaller *d*_tube_. Therefore, due to relatively higher absorption, and contact and Schottky barriers, films comprising smaller *d*_tube_ SWCNTs could be expected to exhibit lower optoelectronic performances in comparison with larger *d*_tube_ ones. However, in our samples these factors are clearly not important, and no correlation is observed. As far as *K*_NORM_ is considered, HWG aerosol CVD SWCNTs of larger *d*_tube_ (1.39–1.60 nm) perform equally well as those with smaller *d*_tube_ (1.13 nm). Furthermore, ferrocene aerosol CVD SWCNTs (blue downward triangles) have generally much larger *d*_tube_ (1.9–2.2 nm) than the HWG aerosol CVD ones (black squares and triangle). Thus, in this comparison, they might be expected to exhibit the highest optoelectronic performance. In reality, however, the opposite is observed, as is evident in Figure 7b. Thus, *d*_tube_ cannot explain the observed variation in *K*_NORM_.

Besides *d*_tube_, also the defect density and the amount of amorphous impurities were observed to vary in our samples. Ideally, due to the sp^2^-hybridized tubular carbon lattice structure, SWCNTs are ballistic electrical conductors [32]. In reality, however, both lattice defects and amorphous impurities are always present to some degree. In this study, the resonant Raman intensity ratios *I*_G_/*I*_D_ were used to gauge both the SWCNT lattice quality and the relative amount of amorphous impurities in the samples. At low *T*_set_ (650–700 °C) conditions, the samples were seemingly contaminated by nongraphitized carbon; while at high *T*_set_ (750–880 °C) they were much cleaner (cf. Figure 1 and Figure 2). Simultaneously, alongside increasing *T*_set_, the *I*_G_/*I*_D_ ratio rises from 5.2 to 48.5 (Figure 4 inset), confirming either better SWCNT lattice crystallinity or decreasing amount of amorphous carbon, or possibly both. Even though this increase was significant, the *I*_G_/*I*_D_ ratios were not predictive for the value of *K*_NORM_ (cf. Figure 4 inset and Figure 7b). In fact, *K*_NORM_ did not show significant changes over the interval from *T*_set_ = 750 °C (*I*_G_/*I*_D_ = 22.0) to *T*_set_ = 880 °C (*I*_G_/*I*_D_ = 48.5), as should be expected if the defect density or amount of amorphous impurities were significant contributors to the performance of the films.

Thus, it seems that neither the average tube diameter *d*_tube_ nor the defect density or amount of amorphous impurities impact the overall optoelectronic performance of the networks significantly. A simple explanation could lie in the very nature of a random SWCNT network, which consists of an enormous number of parallel and series resistors, each resistive component being a single SWCNT, an SWCNT bundle, or a junction. The junction resistances are thought to be on the order of several kilo-ohms, and possibly higher [12], while intratube or -bundle resistances are much lower. Thus, as was discussed before, to a good approximation, the system can be considered to be a resistor network comprising only the junctions. Therefore small or even moderate modifications in the average intrabundle conductivity would not alter the overall network conduction dramatically. Contact and Schottky barriers between SWCNTs, on the other hand, are certainly in principle affected by *d*_tube_. However, where contact barriers are concerned, the distances between individual SWCNTs and bundles likely affect the actual contact resistances much more than the contact barriers alone. In this regard we note that densification by using ethanol is known to reduce the sheet resistance of pristine SWCNT films by about an order of magnitude [6], likely by bringing the network elements closer to each other. Finally, Schottky barriers may not necessarily be important in films consisting of both metallic and semiconducting tubes, since there are always plenty of metallic pathways available for conduction in a dense enough SWCNT network [8]. Charge-transfer doping by acid functionalization [33–34], on the other hand, can affect either the electronic nature of individual tubes [6], barriers between bundles, or both. Again, further work on measuring individual contacts is called for.

## Conclusion

The influence of SWCNT bundle characteristics on the optoelectronic performance of dry-deposited thin films was studied. Bundle length had a profound effect on SWCNT film conductivity, and thus on their performance. Bundle diameters had a lesser effect: thin films comprising larger diameter bundles performed worse than those with smaller diameter ones. However, our analysis indicates that this is due to the higher absorption of thicker bundles, since a geometric normalization of this contribution made the film performances independent of bundle diameter. Finally, neither the defect or impurity density, nor the individual SWCNT diameters affected the overall optoelectronic performances significantly, which thus seems to be best improved by simply increasing the bundle lengths. The bundle diameter, on the other hand, should be minimized, provided bundle lengths can be maximized simultaneously.
